# A sequential, multiple assignment randomized trial comparing web-based education to mobile video interpreter access for improving provider interpreter use in primary care clinics: the mVOCAL hybrid type 3 study protocol

**DOI:** 10.1186/s13012-023-01263-6

**Published:** 2023-03-13

**Authors:** K. Casey Lion, Chuan Zhou, Paul Fishman, Kirsten Senturia, Allison Cole, Kenneth Sherr, Douglas J. Opel, James Stout, Carmen E. Hazim, Louise Warren, Bonnie H. Rains, Cara C. Lewis

**Affiliations:** 1 Department of Pediatrics, University of Washington School of Medicine, Seattle, WA USA; 2 Center for Child Health, Behavior, and Development, Seattle Children’s Research Institute, Seattle, WA 98145-5005 USA; 3 Department of Health Systems and Population Health, University of Washington School of Public Health, Seattle, WA USA; 4 Department of Family Medicine, University of Washington School of Medicine, Seattle, WA USA; 5 Department of Global Health, University of Washington Schools of Medicine and Public Health, Seattle, WA USA; 6 Department of Epidemiology, University of Washington School of Public Health, Seattle, WA USA; 7 Department of Industrial & Systems Engineering, University of Washington, Seattle, WA USA; 8 Center for Clinical and Translational Research, Seattle Children’s Research Institute, Seattle, WA USA; 9 Kaiser Permanente Washington Health Research Institute, Seattle, WA USA

**Keywords:** Interpretation, Language barriers, Limited English proficiency, Healthcare equity, Implementation science, Primary care, Sequential multiple assignment randomized trial (SMART) design, Theoretical Domains Framework

## Abstract

**Background:**

Individuals who use a language other than English for medical care are at risk for disparities related to healthcare safety, patient-centered care, and quality. Professional interpreter use decreases these disparities but remains underutilized, despite widespread access and legal mandates. In this study, we compare two discrete implementation strategies for improving interpreter use: (1) enhanced education targeting intrapersonal barriers to use delivered in a scalable format (interactive web-based educational modules) and (2) a strategy targeting system barriers to use in which mobile video interpreting is enabled on providers’ own mobile devices.

**Methods:**

We will conduct a type 3 hybrid implementation-effectiveness study in 3–5 primary care organizations, using a sequential multiple assignment randomized trial (SMART) design. Our primary implementation outcome is interpreter use, calculated by matching clinic visits to interpreter invoices. Our secondary effectiveness outcome is patient comprehension, determined by comparing patient-reported to provider-documented visit diagnosis. Enrolled providers (*n* = 55) will be randomized to mobile video interpreting or educational modules, plus standard interpreter access. After 9 months, providers with high interpreter use will continue as assigned; those with lower use will be randomized to continue as before or add the alternative strategy. After another 9 months, both strategies will be available to enrolled providers for 9 more months. Providers will complete 2 surveys (beginning and end) and 3 in-depth interviews (beginning, middle, and end) to understand barriers to interpreter use, based on the Theoretical Domains Framework. Patients who use a language other than English will be surveyed (*n* = 648) and interviewed (*n* = 75) following visits with enrolled providers to understand their experiences with communication. Visits will be video recorded (*n* = 100) to assess fidelity to assigned strategies. We will explore strategy mechanism activation to refine causal pathway models using a quantitative plus qualitative approach. We will also determine the incremental cost-effectiveness of each implementation strategy from a healthcare organization perspective, using administrative and provider survey data.

**Discussion:**

Determining how these two scalable strategies, alone and in sequence, perform for improving interpreter use, the mechanisms by which they do so, and at what cost, will provide critical insights for addressing a persistent cause of healthcare disparities.

**Trial registration:**

NCT05591586.

Contributions to the literature

- Our study examines two scalable approaches for improving professional interpreter use, which previous research has found to be both effective for decreasing healthcare disparities and underutilized in most clinical settings.
- Our study will examine interpreter use and patient comprehension as outcomes and will be the first study to explore, in detail, the mechanisms by which the intervention strategies achieve those outcomes.
- Our study is designed to provide leaders of clinical organizations the information they need, including a cost-effectiveness analysis, to select the strategy to most effectively improve interpreter use and decrease language-based disparities in their particular contexts.

## Background

Effective communication is essential for safe and equitable care. Twenty-five million people in the United States of America (USA) report speaking English less than “very well” and, as a result, have limited access to safe and high-quality medical care [1, 2]. Language barriers in healthcare are associated with lower patient comprehension, adherence, and satisfaction [3–6]; higher costs, longer hospital stays, and increased odds of readmission [7–11]; less treatment for pain [12, 13]; increased risk of serious adverse events [14–17]; and increased mortality [18]. Given the importance of effective communication to high-quality medical care, improving communication with patients who use a language other than English for medical care has been named a national priority [19].

### The research-to-practice gap: underuse of interpretation is a persistent problem

Interpretation provided by trained medical interpreters, whether in person or via telephone or video, has repeatedly been shown to mitigate disparities in care for patients with language barriers [20, 21]. However, despite clear evidence of benefit, wide availability, and federal, state, and regulatory mandates requiring professional interpreter use for patients who use a language other than English [22, 23], underuse remains pervasive [24–32]. Nearly half of US pediatricians report using no professional interpreters with families who use a language other than English [33]. Interpreter use in acute care settings is similarly low, with 17–45% of patients receiving any [24–27]. Providers often use English or untrained ad hoc interpreters (family or friends), a practice associated with clinically important errors up to 77% of the time [28, 29, 34–36].

Barriers to interpreter use exist at multiple levels, with evidence that providers weigh barriers against anticipated benefit for every communication [36]. Commonly identified barriers map onto the Theoretical Domains Framework (TDF) [37], which integrates behavior change theories for application in health services and implementation research. These include *provider-level barriers* such as conceptual and technical knowledge (uncertainty about need for or how to access interpreters), beliefs about capabilities (lack of confidence in interpreter use, belief their own non-English language skills are adequate), beliefs about consequences (uncertainty of benefit, anticipated frustration), and environmental context (time pressure); *team-level barriers* including social influences (a culture of “getting by” without an interpreter); and *system-level barriers* including environmental context and resources (difficulty identifying patients with language barriers and lengthy or difficult processes to access interpreters) [28, 38–42]. While in-person interpreters are preferred by providers [39, 43–45], remote methods have benefits, such as being widely accessible, immediately available, and the only option for uncommon languages. Among remote methods, video costs more than telephone but is often preferred by providers [45–48].

### Previously studied strategies lack attribution, scalability, and data on costs and mechanisms

Strategies to improve interpreter use generally fall into three categories: provider education (focused on provider-level barriers), systems improvements (focused on system-level barriers and the provider-system interaction), and multifaceted, multilevel interventions. Provider education is most common, typically delivered via in-person workshops [49–52]. Though such trainings typically improve knowledge and confidence, it is unknown whether such improvements lead to improved interpreter use [49, 51, 52]. Systems interventions aim to make access easier or offer access to preferred interpreter types, such as installing dedicated bedside interpreter phones with 1-touch dialing [53] or enabling access to shared video interpreter units [36]. Systems interventions have generally yielded only modest improvement [54–56], likely because important barriers have remained: improving access to telephone interpretation did not address provider dislike for it, and current models for video interpreter use involve shared devices (e.g., clinic laptop), which introduce barriers around finding and using it. Multifaceted, multilevel interventions, combining education, systems interventions, and facilitation, have been most successful [54, 57–59], yet such approaches are time and resource intensive and lack data on which aspects were most effective [60, 61]. No studies have yet considered mechanisms of action, few have measured cost, and many interventions are not scalable.

### Preliminary studies

Our previous work showed the effectiveness of video interpretation for improving communication with families with a language barrier [46]. In a randomized clinical trial enrolling 249 Spanish-speaking families in an emergency department, we found that assignment to video interpretation, compared to telephone, was associated with significantly higher interpreter use [36], parent understanding [46], and provider satisfaction [48]. However, half of video-recorded interactions still did not use a professional interpreter, and 43% of providers reported trouble accessing an interpreter. These_findings support video interpretation as an effective evidence-based practice for communicating across language barriers, but without an optimized platform or strategy for engaging with it.

We therefore explored the feasibility and acceptability of mobile video interpreting on personal devices, as a novel strategy to deliver the evidence-based practice of video interpretation. Mobile video interpreting overcomes barriers associated with conventional access via shared devices [39, 47, 62]. To determine its feasibility and acceptability in primary care, we conducted 6 simulated patient sessions with mobile video interpreting and then interviewed the provider. Providers were universally positive about it, with scores on the acceptability of intervention measure and feasibility of intervention measure of 4.7 and 4.9 out of 5 [63]. We also surveyed a panel of 67 PCPs in our region to assess mobile video interpreting acceptability in practice. Most (71%) said they would be “very likely” or “somewhat likely” to use mobile video interpreting if offered. These results support mobile video interpreting as an acceptable and potentially feasible strategy for accessing the evidence-based practice of professional video interpreter use.

### Study aims

To address current knowledge gaps, we will test two implementation strategies for improving interpreter use in primary care and examining implementation and effectiveness outcomes, cost-effectiveness, and mechanisms of action. Providers will be enrolled and randomized to one of two strategies, alone or in sequence, using a Sequential Multiple Assignment Randomized Trial (SMART) design [64–66]. One strategy, web-based educational modules, targets known deficits in provider knowledge, confidence, and motivation around interpreter use. The second strategy, mobile video interpreting, provides quick access to video interpretation. Providers are more likely not only to use video interpretation versus telephone [46, 47] but also mobile video interpreting overcomes system-level barriers to shared device use, as providers will access professional video interpreters on their own smartphone or tablet. Data will be collected from enrolled providers and their patients/families who use a language other than English, via administrative data, surveys, qualitative interviews, and video-recorded clinic visits.

Our specific aims are as follows:

- Aim 1: To compare the effectiveness of two implementation strategies, alone and in combination, to improve use of interpretation and comprehension for patients/parents who use a language other than English, seen in adult and pediatric primary care settings
- Aim 2: To explore mobile video interpreting and education implementation strategies’ ability to activate putative provider-level mechanisms
- Aim 3: To determine the incremental cost-effectiveness from a healthcare organization perspective of each implementation strategy (mobile video interpreting, education, both)

## Methods

### Conceptual model

**Fig. 1:**
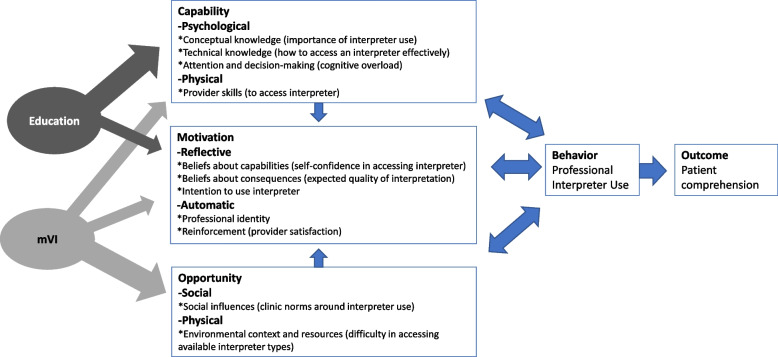
Conceptual model depicting relationships between determinants, behavior, and outcomes related to professional interpreter use, based on the Theoretical Domains Framework as mapped to the Behavior Change Wheel’s COM-B system. Each barrier has one example listed; additional barriers are in Table 1. Expected areas of effect with our two implementation strategies are depicted on the right; wider arrows represent larger expected effects

The TDF, mapped to the Behavior Change Wheel’s COM-B (capability, opportunity, motivation—behavior) system, informed our conceptual model (Fig. 1) [37, 67]. The TDF is an integrative theoretical framework that has been used across healthcare settings to inform implementation strategies, especially those requiring behavior change [68, 69]. It underwent rigorous refinement using discriminant content validation and fuzzy cluster analysis and was then mapped to the COM-B system to provide theory-based relationships between the barriers laid out in the TDF. In our conceptual model, we identify relevant TDF domains for each of the COM-B’s major categories as contributing to the target behavior, interpreter use. These COM-B categories are capability, divided into psychological, which includes knowledge and decision-making, and physical, which includes skills for interpreter access; motivation, divided into reflective, including provider beliefs about their abilities and the consequences of their decisions, and automatic, which includes professional identity and positive reinforcement; and opportunity, divided into social, which includes clinic interpreter use norms, and physical, which includes environmental context and resources, such as current interpreter access (see Table 1 for detailed list). We expect both of our study strategies to influence provider capability and motivation to use interpreters, but we expect education to influence provider capability more markedly and mobile video interpreting to have a strong and unique influence on opportunity.

**Table 1:** Study measures and data sources

		**Data source**						
**Variable**	**Definition**	Pro Svy	Pro Int	Pt Svy	Pt Int	Video	EMR	Admin
**Implementation and effectiveness outcomes**								
Interpreter use (primary outcome)	*Dichotomous variable* per non-English language patient visit, constructed by matching professional interpreter vendor invoices to clinic visits with enrolled providers; calculated overall and as assigned (e.g., mobile video interpreting for assigned providers) [27, 46, 57]							X
Patient/parent comprehension	*Dichotomous variable* coded as yes vs no or unclear, based on 2 blinded coders evaluating concordance between patient-reported and provider-documented diagnosis, against the standard of whether a follow-up provider would know the diagnosis based on the patient-reported information [46, 70]			X			X	
**Patient factors**								
Patient/parent health literacy	Confidence in filling out medical forms, with options from extremely confident (0) to not at all confident (4); scores ≥ 2 considered low health literacy [71–73]			X				
Visit type	Annual/wellness exam, acute visit, or chronic condition follow-up						X	X
Patient demographics	*-Insurance:* None/public insurance, private insurance *-Race/ethnicity:* American Indian or Alaska Native, Asian, Black, Hispanic, Native Hawaiian or Pacific Islander, multiracial/other, White, prefer to self-describe *-Sex*: Male, female, or self-describe *-Age*: Continuous variable			X				X
Respondent demographics (patient or parent)	*-English proficiency*: Very well, well, not well, not at all *-Education*: < high school, high school graduate, > high school *-Respondent relationship to patient*: Self, mother, father, other guardian			X				
**Provider and clinic barriers, organized by Theoretical Domain Framework and COM-B categories** [37]								
**Capability—psychological**								
Conceptual knowledge	Importance of communication and interpreter use, via TDF questionnaire [74] and interview	X	X					
Technical knowledge	How to access and use an interpreter effectively, via TDF questionnaire [74], interview, and coded provider behaviors on video recording	X	X			X		
Attention and decisions	Cognitive overload, via interview		X					
**Capability—physical**								
Provider skills	Skills to access interpreter and communicate effectively, via TDF questionnaire [74], interview, and coded provider behaviors on video recording	X	X			X		
**Motivation—reflective**								
Beliefs about capabilities	Confidence in ability to access and communicate via interpreter, via TDF questionnaire [74] and interview	X	X					
Beliefs about consequences	Belief that interpreter use is important; expected barriers, delays, difficulties; expected quality of communication and interpretation; via TDF questionnaire [74] and interview	X	X					
Goals and intentions	Intention to use interpreter via TDF questionnaire [74] and interview and engagement with assigned strategy, measured by mobile video interpreting use and module completion	X	X					X
**Motivation—automatic**								
Professional identity	Sense of professional duty to use interpretation, via TDF questionnaire [74]	X						
Reinforcement	Provider-reported satisfaction and communication quality via interview; patient-reported satisfaction with communication and interpretation, via survey (the Consumer Assessment of Healthcare Providers and Systems (CAHPS) communication composite [75–78] and the interpreter satisfaction questionnaire[79]) and interview		X	X	X			
**Opportunity—social**								
Social influences	Group and leadership norms, via TDF questionnaire [74], Organizational Readiness for Implementing Change (ORIC) questionnaire [80], the Implementation Leadership Scale (ILS) [81], and interview	X	X					
**Opportunity—physical**								
Environmental context and resources	Time constraints, types of interpretation available, difficulty in accessing available interpreter types, wireless infrastructure, and clinic process to identify LEP patients, via TDF questionnaire [74], interview, and coded provider behaviors on video recording	X	X			X		

*Pro Svy*, provider survey; *Pro Int*, provider interview; *Pt Svy*, patient/parent survey; *Pt Int*, patient/parent interview; video, video-recorded visits; *EMR*, electronic medical record; *Admin*, administrative data; *TDF*, Theoretical Domains Framework

### Study design and randomization

This type 3 hybrid implementation-effectiveness study will test two discrete implementation strategies for improving professional interpreter use (primary implementation outcome) and patient comprehension (secondary effectiveness outcome) in primary care. The implementation strategies—interactive web-based educational modules and access to mobile video interpreting—target different sets of barriers to professional interpreter use, an evidence-based practice [20, 21, 34, 82, 83]. Our results will therefore provide insights into how best to promote implementation of a well-studied, well-established practice known to improve outcomes but inconsistently used. As the potency of barriers may vary by provider and clinic, we will test the strategies alone and in combination, using a SMART design, with provider-level randomization.

**Fig. 2:**
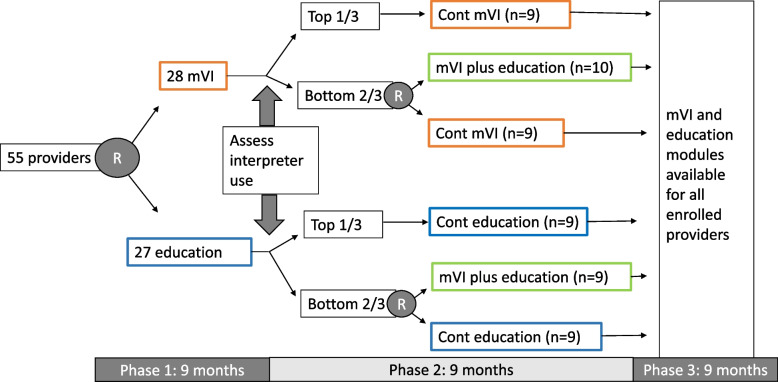
SMART design depicting 2 rounds of provider randomization over 18 months with active data collection and 9 months of follow-up

A total of 55 providers from 3 to 5 primary care clinical organizations will be randomized 1:1 to either education or mobile video interpreting access, stratified by baseline interpreter use and clinic (phase 1; Fig. 2). Randomization will occur within REDCap, using a sequence generated by the study biostatistician and implemented by a research coordinator. After 9 months, providers with interpreter use in the top tertile (within strategy) will remain with the original strategy; those in the bottom two tertiles will be randomized 1:1 again, to continue the original strategy or to add the second strategy to the first (phase 2). After another 9 months of data collection, we will provide free access to both mobile video interpreting and educational modules to all enrolled providers and then track voluntary uptake by those not previously exposed for another 9 months (phase 3). Data collection will include *administrative data* to track interpreter use (primary outcome); *patient surveys and qualitative interviews* to determine diagnosis comprehension (secondary outcome) and communication quality; *provider surveys and qualitative interviews* to assess contextual and intrapersonal barriers and moderators; and *visit video recording* to capture additional barriers and determine fidelity of strategy implementation. We will assess each strategy’s effectiveness, alone and in combination, for improving professional interpreter use and patient comprehension. We will explore mechanisms by which these strategies work and evaluate the relative strategy-specific costs.

### Implementation strategies

Our selected implementation strategies target primarily intrapersonal barriers to interpreter use, although mobile video interpreting does so by altering the environment and resources (i.e., opportunities) available to that provider [37, 84]. Strategy assignment will thus happen by individual provider. However, knowing the importance of team, clinic, and patient-level factors for influencing provider behavior, we will also capture data at these levels. Detailed strategy specification, following Proctor’s recommendations [85, 86], is presented in Table 2.

**Table 2:** Detailed implementation strategy specification, following Proctor’s recommendations

Name it	Define it	Specify it					
		**Actor**	**Action**	**Action target**	**Temporality**	**Dose**	**Implementation outcomes affected**
Name the strategy	Define the implementation strategy and any discrete components operationally	Who delivers the implementation strategy?	What action takes place to support implementation?	Who or what receives the actions?	When or at what phase are the actions delivered?	At what frequency and intensity are the actions conducted?	What specific implementation outcome is the strategy aiming to affect?
**Wed-based educational modules**	Provision of ten interactive web-based adult-learning-oriented educational modules	Research team	Distribute educational module access links and reminders to providers to encourage access to existing and new modules	-Healthcare providers serving patients with a language other than English -Provider motivation and capability to access and use interpreters, conceptual and technical knowledge and confidence around interpreter use, and intention to use an interpreter	Educational modules shared in initial 9-month implementation phase intervals, with ongoing access	New 5–15-min-long modules released weekly with continuous access following release	-Increased acceptability of interpreter use among providers -Increased provider adoption (uptake) of interpreter services with patients who use a language other than English -Increased penetration of interpreter use among providers and within healthcare setting -Increased appropriateness (perceived relevance) of interpreter services with patients who use a language other than English -Increased fidelity with which providers access and use interpreters in accordance with published guidance
	Clinic-specific interpreter information		Distribute clinic-specific interpreter information and tips to providers		Distribution occurs at beginning of initial 9-month implementation phase	Distributed once	-Increased provider adoption of interpreter services -Increased penetration of interpreter use among providers and within healthcare setting
	Feedback to enrolled providers		Share reports on interpreter use to enrolled providers		Report sharing provided across all 9-month implementation phase intervals	Report shared quarterly	-Increased provider adoption of interpreter services -Increased penetration interpreter use among providers and within healthcare setting -Increased sustainability of interpreter use as part of normal operations within the healthcare setting -Increased appropriateness (perceived relevance) of interpreter services with patients who use a language other than English
Mobile video interpreting access	Access to mobile video interpreting via app on smart phone	Research team	Provide mobile video interpreting application access to providers for use with patients who use a language other than English for medical care	-Healthcare providers serving patients who use a language other than English -Provider opportunity, motivation, and capability to access and use interpreters, context in which providers decide about interpreter use, environment, and resources to interpreter access	Mobile video interpreting access provided across all 9-month implementation phase intervals	Continuous access to mobile video interpreting provided	-Increased feasibility of accessing interpretation services due to mobile application -Increased acceptability of interpreter use among providers -Increased provider adoption (uptake) of interpreter services with patients who use a language other than English -Increased penetration of interpreter use among providers and within healthcare setting -Reduced cost of in person interpretation
	Tip sheet for app use best practices		Written information regarding app access and best practices for use, including positioning and troubleshooting		Distribution occurs at beginning of initial 9-month implementation phase	Distributed once	-Increased fidelity with which providers access and use interpreters in accordance with published guidance -Increased appropriateness (perceived relevance) of interpreter services with patients who use a language other than English -Increased provider adoption of interpreter services -Increased penetration of interpreter use among providers and within healthcare setting -Increased feasibility of accessing interpretation services
	Technical support		-Provide technical support as needed -Send email reminders of technical support availability		Technical support access provided across all 9-month implementation phase intervals	Continuous access to technical support provided Email reminders of technical support availability sent weekly for the first month and monthly afterwards	-Increased feasibility of accessing interpretation services via mobile application -Increased acceptability of interpreter use via virtual platform
	Feedback to enrolled providers		Share reports on interpreter use to enrolled providers		Report sharing provided across all 9-month implementation phase intervals	Report shared quarterly	-Increased provider adoption of interpreter services -Increased penetration of interpreter use among providers and within healthcare setting -Increased sustainability of interpreter use as part of normal operations within the healthcare setting -Increased appropriateness (perceived relevance) of interpreter services with patients who use a language other than English

*Note*: Term “initial implementation phase” denotes the first 9-month interval that a provider receives the implementation strategy in question. It does not signify the first 9-month interval of the entire trial

#### Web-based educational modules

The education implementation strategy will consist of six 10- to 15-min web-based modules, a tip sheet with clinic-specific interpreter access and use information, and four 5-min booster modules, all delivered online, along with quarterly reports on interpreter use to the enrolled provider. Education aims to improve provider motivation and capability related to interpreter use, by increasing conceptual and technical knowledge, enhancing interpreter access skills, shifting beliefs about their own capabilities and the consequences of use or nonuse, and increasing the intention to use an interpreter.

The educational module content is based on Seattle Children’s Hospital’s rigorously developed in-person workshop series, CONNECTing Through Interpreters [49–52]. In partnership with the interactive Medical Training Resources (iMTR) group at the University of Washington (UW; depts.washington.edu/imtr/) and content experts including experienced interpreters and providers, we transformed the workshops into interactive web-based modules. Modules were pilot tested with 15 primary care providers (PCPs) and revised based on feedback. Module-assigned providers will view them at a time and place they choose. We will track when participants access and complete modules as a marker of engagement.

The *online modules* cover 5 topics: (1) importance and fundamentals of good communication (delivered in 2 modules), (2) importance of professional interpreter use and disparities for populations with language barriers, (3) how to use an interpreter effectively, (4) what to do when the interpreted encounter is not going well, and (5) remote interpreter use and system’s challenges. Each module is 10–15 min long with audio, visual, and video content, developed using best practices from adult learning theory. Providers will be prompted to view a new module each week until all have been viewed.

During months 3–6 post-randomization, 4 brief (5 min) *booster modules* will be released, reviewing crucial points from initial modules. Boosters have been found to support behavior change in other settings [87]. Weekly reminders will be sent until they are complete. Providers who complete all modules will be eligible for points for continuing medical education (CME) and/or Maintenance of Certification (MOC); these points must be earned to maintain medical licensure and board certification and thus provide incentive for completion.

The *clinic-specific interpreter access and use information* will be distributed via email. This sheet will include instructions for accessing interpreters in their clinic via the normal process, including the vendor phone number, tips for using the clinic telephones (e.g., how to adjust the speakerphone volume), ideas for streamlining the process, where shared equipment is stored, and how to report problems.

*Feedback to enrolled providers* will be provided quarterly with both strategies, as a report of the percent of visits with patients who use a language other than English for which the provider used professional interpretation.

#### Mobile video interpreting access

The mobile video interpreting access strategy will provide access to mobile video interpreting, technical support, a tip sheet for mobile video interpreting use, and an extra charger, shock-resistant case, disposable antimicrobial sleeves, and a positioning stand to support clinical use of the provider’s own device, along with quarterly reports on the enrolled provider’s interpreter use. Mobile video interpreting-assigned providers can use a study-issued smartphone instead of their own. The mobile video interpreting strategy aims to improve provider motivation, capability, and opportunity related to interpreter use, by decreasing cognitive overload, enhancing interpreter access skills, shifting provider beliefs about capabilities and the consequences of interpreter use, reinforcing use via satisfaction, and altering the environmental context and resources to make access easier and use more rewarding (Table 2).

*Access to mobile video interpreting* is achieved by downloading the application (app) online and then entering an access code linked to a billing account; after being entered, the code is no longer visible. Access can thus be controlled by study staff. Study staff will download and orient providers to the app, demonstrate use, and answer questions. *Technical support* will be offered on demand. A *tip sheet* will be emailed that includes mobile video interpreting instructions and best practices.

Several interpretation vendors have similar apps that can be downloaded onto personal devices but are rarely used in this way. These apps are HIPAA compliant, use end-to-end encryption, and are accessed with one touch (i.e., no additional log in or passwords); no data is downloaded to the device.

*Feedback to enrolled providers* will be provided quarterly with both strategies, as a report of the percent of visits with patients who use a language other than English for which the provider used professional interpretation.

### Study populations and setting

#### Providers

We will enroll 55 PCPs from 3 to 5 primary care organizations in Washington state. These organizations will include both academically affiliated and nonacademic sites and vary in terms of leadership and governance structures. Clinics will enroll based on provider interest, but each provider will choose whether to enroll. Eligible providers will practice at the enrolled clinic at least 40% time and see at least 7 patients requiring interpretation per month, on average. If the provider is proficient in a non-English language, they will see at least 7 patients per month who use a different language (in which they are not proficient). We will enroll and initially randomize 55 providers, to retain 47 through the second interview and 40 through the third (73% retention; see next section for sample size considerations).

#### Patients

We will enroll 3 populations of adult patients or parents of pediatric patients (henceforth “patients”) who use a language other than English, all being seen by enrolled providers. For our *administrative population*, we will include administrative data from all patients who were recorded as using a language other than English in the medical record and were seen by enrolled providers, for the interpreter use outcome. For our *survey population*, we will enroll patients who prefer medical care in the four most common non-English languages across clinics, who are in clinic for an acute concern (e.g., sore throat, new ankle pain). These individuals will be invited to complete a survey (*n* = 648), and a subset will be invited to complete a 20–30 min qualitative interview (*n* = 75). We will also recruit patients for our *video-recording population* (*n* = 100). Patients who use a language other than English with any visit type who consent will be eligible for video recording.

### Data collection, study measures, and sample size

Outcome measures include our primary implementation outcome of interpreter use and our secondary effectiveness outcome of patient/parent comprehension. Additional measures related to organizational context, provider-reported barriers and facilitators of interpreter use, and intervention fidelity are laid out in Table 1.

#### Interpreter use

Interpreter vendor invoices will be collected from companies that clinics currently contract with; mobile video interpreting invoices will be managed by the study team. All professional interpreter invoices (not just mobile video interpreting) will be matched to clinic visits for patients who use a language other than English (all languages) for enrolled providers. We will calculate baseline interpreter use for enrolled providers for the six months pre-randomization and then randomize 1:1 to education or mobile video interpreting, stratified by baseline use and clinic. We will calculate interpreter use, both overall and strategy consistent, continuously throughout phases 1–3; other data collection will end after phase 2.

For analysis, interpreter use will be defined as a dichotomous variable at the level of the clinic visit. Visits with patients who use a language other than English with any billed professional interpreter use will be coded as “yes,” and the remainder will be coded as “no.”

Sample size calculations consider aim 1 group comparisons (mobile video interpreting, education, combination) at the end of phase 2. We assume loss of up to 9 providers (e.g., to job change; 16%) over the study; we expect attrition (up to 27%) in provider interviews and surveys, but that will not impact aim 1 power. With 5796 total encounters with patients who use a language other than English (7 visits/provider/month), we expect 1932 non-English visits per group, which will provide > 80% power to detect a 5% difference in proportion of professionally interpreted visits by groups [46, 57]. This will be readily feasible with administrative data.

#### Patient/parent comprehension

Patient comprehension will be determined by asking surveyed patients (*n* = 648) to report the diagnosis they received during their visit with an enrolled provider. The parent-reported diagnosis will then be compared to the provider-documented diagnosis, which trained abstractors will have abstracted from the EMR. Two coders blinded to study assignment will compare the documented diagnosis to the patient-reported diagnosis to determine comprehension, coded as yes, concordant; no, not concordant; or unclear, based on the standard of whether a different follow-up provider would likely know the diagnosis based on the information provided by the patient. For analysis, comprehension will be coded as yes or no/unclear. We have successfully used these procedures previously [46].

In addition to measuring comprehension, the survey will use validated measures to collect demographics and satisfaction with communication and interpretation. The tablet-based survey will have an audio feature to allow patients to read or hear the questions in 4 non-English languages. The survey will be completed in the clinic whenever possible; otherwise, the patient will complete it within 7 days, independently online or over the telephone with a bilingual research coordinator or professional interpreter.

Based on aim 1 analyses, with 216 completed patient surveys per group (648 total), we will have ≥ *80% power to detect a 14% difference in diagnosis comprehension by group* [46]. This will also be feasible, achieved by surveying 7–12 patients per clinic per month for 18 months.

#### Provider attributes and organizational context

These data will be collected via 2 surveys and 3 interviews over the course of the study. Providers will complete a web-based survey at baseline, before initial randomization, to assess demographics and barriers to interpreter use via the TDF Questionnaire, [74] Organizational Readiness for Implementing Change (ORIC) questionnaire [80], and the Implementation Leadership Scale (ILS) [81]. We will repeat the survey at the end of phase 2, to capture changes over time and provider time and costs associated with the implementation strategies.

**Fig. 3:**
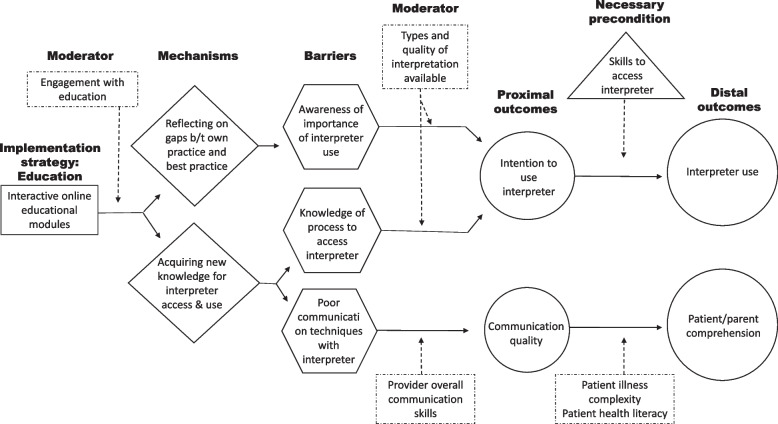
Preliminary causal pathway diagram for mechanisms associated with education as a strategy

**Fig. 4:**
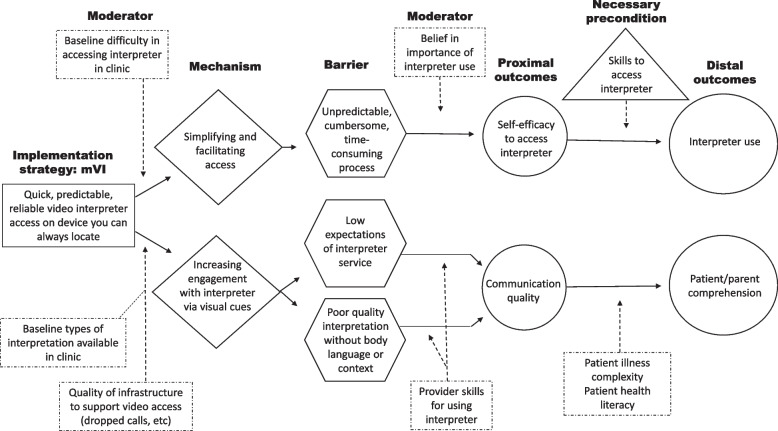
Preliminary causal pathway diagram for mechanisms associated with mobile video interpreting as a strategy

Enrolled providers will also complete qualitative interviews (1) before initial randomization, (2) during phase 1, and (3) during phase 2. Interviews will explore contextual and personal factors that serve as barriers, moderators, mechanisms, and proximal outcomes of interpreter use (see Figs. 3 & 4 for preliminary causal pathway diagrams). We will use qualitative interviews given the lack of survey measures for many factors, and concern for social desirability bias, as providers may not endorse interpreter nonuse on surveys but may be more likely to in the context of a conversation. Provider qualitative and quantitative data will be analyzed together (see “Data analysis”).

#### Patient communication experiences

A subset of patients completing the survey will be invited to complete a 30-min qualitative interview [88]. Survey respondents who endorse having a concern about how their provider communicated with them will be invited to interview [89], as will a random sample of others (total *n* = 75). Our goal is to understand how communication occurred during the visit, how effective the patient found that communication to be and why, and the details of any concerns the patient had. The interview will be completed in the clinic prior to departure whenever possible; otherwise, the patient will have 7 days to complete it, over the telephone with a bilingual research coordinator or via professional interpreter, in one of our 4 eligible non-English languages.

We estimated initial qualitative sample size based on the heterogeneity of our target group, the number of research sites, and the complexity of the areas of inquiry. The initial sample estimates will be adjusted as needed to achieve data sufficiency [90].

#### Video recording

Video-recorded visits with patients who use a language other than English (*n* = 100) will provide granular, objective data regarding interpreter use, technical difficulties, communication delays, and provider use of best-practice techniques for communicating with an interpreter, to supplement provider- and patient-reported data. Trained coders will code videos for specific behaviors, based on the coding scheme developed previously [36], to provide data on barriers, mechanisms, proximal outcomes of interpreter use, and strategy fidelity (Table 1). The video recording sample size is based on our previous work and logistical considerations, with 100 recordings both feasible and likely to achieve data sufficiency.

#### Cost data

Administrative cost data collected from clinics will include costs associated with interpreter vendor invoices and contracts; interpreter-specific clinic hardware (e.g., dedicated speakerphones); wireless Internet; and educational module development, following recommendations for economic analysis in implementation science [91]. Provider-incurred time and costs will be collected via the final survey, including time spent on each strategy, excess data charges associated with mobile video interpreting use (if any), and wear or damage to personal devices. Study team time related to implementing each strategy (e.g., installing mobile video interpreting, reminder emails) will be tracked in real time, as they would be performed by clinic staff with real-world implementation. We do not expect changes in clinic visit length, based on time-motion studies of interpreted patient visits [92, 93].

### Data analysis

Primary quantitative analyses will be conducted using an intention-to-treat approach. Provider and patient characteristics will be summarized overall and by strategy. Missing data will be minimized through communication with participants regarding the importance of completing surveys and interviews, participant incentives, offering multiple languages and modalities for survey and interview completion, and completing surveys and interviews on-site when possible. For our primary outcome, we expect interpreter invoice data to be complete, given our previous experience [12, 46, 57]. We will track interpreter use for all enrolled providers for the entire study, even if they do not complete interviews or surveys. For our secondary outcome, diagnosis comprehension, patterns of data missingness will be examined. We expect randomization will help protect against imbalance in unobserved confounders, so our main concern will be with missing data. We will conduct sensitivity analyses based on multiple imputations to assess the impact of missing data, in which we will generate multiple imputed datasets with missing values imputed by pooling information from observed data, and then combine statistical inferences across the multiply-imputed datasets [94–96].

#### Aim 1: Compare the effectiveness of two implementation strategies, alone and in combination, to improve use of interpretation and comprehension for patients/parents with language barriers seen in adult/pediatric primary care settings

We hypothesize that, compared to educational modules, provider access to mobile video interpreting will lead to (*H1*) greater odds of interpreter use for visits with patients/parents with language barriers (primary outcome) and (*H2*) better comprehension among patients/parents with language barriers. We also hypothesize (*H3*) that mobile video interpreting and educational modules together will yield greater odds of interpreter use than either strategy alone.

To test *H1* and *H3*, we will use assigned strategy and data collected during phases 1 and 2. Under the SMART design, comparisons of first-stage interventions, comparisons of second-stage interventions, and comparisons of the adaptive intervention with both stages can be conducted simultaneously using standard software with a technique called a “weighted and replicated” regression approach, using weighted generalized estimating equations (GEE) [66, 97]. Weighted GEE allows us to work with binary outcomes and weights and adjust for clustering within providers. Within-clinic correlations will be assessed by including clinic-specific random effects in our regression models and estimating the intra-cluster correlation coefficients. Significance of the intra-cluster correlation coefficients will be examined by comparing models with and without clinic-specific random effects using likelihood ratio tests. If no strong within-clinic correlation is detected, we will use fixed-effects regression models for their better power; otherwise, estimates and inference based on random-effects regression models will be reported. *H1* and *H3* will be tested using the Wald test and robust standard error estimates [66, 97]. Model-based estimates of odds ratio comparing education to mobile video interpreting or both will be reported, along with 95% confidence intervals [98].

To test *H2*, our analytic sample will include only patients who completed a post-visit survey (*n* = 648). A weighted GEE logistic regression model predicting patient/parent comprehension at the visit level will be estimated. Baseline covariates will include the clinic, patient demographics (age, sex, language), and patient comorbid conditions [99–102], pooled at the provider level. Model-based estimates of the odds ratio comparing education to mobile video interpreting or both will be reported, along with 95% confidence intervals computed via parametric bootstrapping [98].

#### Aim 2 Explore mobile video interpreting and education implementation strategies’ ability to activate putative provider-level mechanisms

We predict that implementation via mobile video interpreting will activate mechanisms that are more directly and strongly linked to provider behavior, while education’s mechanism activation will more often affect intrapersonal barriers without changing behavior.

We will use a quantitative plus qualitative approach to explore putative mechanisms, where both are analyzed together to understand data in context [88]. Interviews will be audio-recorded, transcribed, translated as appropriate, and reviewed for accuracy. Using an iteratively developed codebook, we will code all data stratified by interpreter use and TDF attributes, upload data into Dedoose Version 9.0.17 for thematic analysis [103–105], and use the 6 analysis steps outlined by Braun and Clarke [106]. Data synthesis will be conducted from code reports utilizing an annotation and tabular system. We will analyze provider and patient data separately.

Video-recording analysis will be based on our previously developed coding scheme [36], with modifications based on coding the first 5 videos. We expect coding to include communication/interpretation method, duration, interpretation technical difficulties (e.g., dropped calls), interpreter or device positioning in room, provider use of jargon and acronyms, and clarifications between provider and interpreter. Initial videos will be double coded, until kappa statistics for interrater reliability are greater than 0.75. Subsequent videos will be single coded, with a random 10% double coded. Fidelity to assigned strategy will be defined as use of mobile video interpreting for assigned providers and use of best practices for communicating through an interpreter for education-assigned providers.

Qualitative analysis of interviews and video recordings will occur with reference to provider quantitative data, for example, by interpreter use (high vs low) and survey-reported TDF attributes, following NIH guidelines for mixed-methods best practices [107]. Provider interviews and videos will be considered as a set, to assess for changes over time, by assigned strategy. The relationships we investigate will be guided by preliminary causal pathway models (Figs. 2 & 3). These models, developed with best available evidence, lay out the putative mechanisms of each implementation strategy, including organizational and intrapersonal moderators, specific barriers, and proximal and distal outcomes. In this approach, we will explore hypothesized relationships and invite emergent mechanisms we had not previously considered given this work’s exploratory nature. Little is known about the mechanisms by which particular strategies influence interpreter use or even if things like acquiring facts serve as mediators on the pathway from strategy to outcome [108, 109]. Per Kazdin, identifying mediators and mechanisms of change allows greater reason and parsimony in selecting implementation strategies and should allow attainment of greater improvements over time as we understand exactly how improvement occurs [110]. We will refine our causal pathway diagrams and generate new ones reflecting the evidence gathered through this study.

#### Aim 3: Determine the incremental cost-effectiveness from a healthcare organization perspective of each implementation strategy (mobile video interpreting, education, and both)

We hypothesize that, relative to educational modules, mobile video interpreting will be more cost-effective (*H4a*) per additional interpreted clinic visit and (*H4b*) per additional instance of patient comprehension.

The estimated incremental cost-effectiveness ratios (ICER) will provide evidence of the resources required to increase interpreted clinic visits and improve patient comprehension [111]. Our goal is to support decision-making about which strategy healthcare organization leaders may choose to implement, and thus, we will estimate ICERs from the organization perspective.

Effectiveness measures will be based on Aim 1 analyses; cost data will come from two sources. The first source is administrative, including vendor invoices and budgets for payroll. Costs that cannot be determined will be estimated with a micro-costing approach in which unit cost multipliers are applied to the quantity of each type of service or resource utilized; examples include the use of shared resources (space, office equipment) and opportunity costs experienced by clinic staff. All cost data are summed to obtain total costs [112], using an approach we have used previously [113, 114]. While mobile video interpreting-assigned providers may also have used other professional interpretation, we will assign mobile video interpreting-related costs to the mobile video interpreting and combination groups and nonmobile video interpreting interpreter costs (which would not be necessary if a clinic used mobile video interpreting only) to the education group. Interpreter costs will be based on actual usage from vendor invoices, attributed to assigned group. Education module development will be annuitized over the study period. Time costs for providers (time on modules, learning to use mobile video interpreting) and study staff (reminder emails, mobile video interpreting support) will be estimated using the mean hourly wage from the National Compensation Survey, plus fringe rates from the Bureau of Labor Statistics Employer Costs for Employee Compensation. Provider costs due to own-device use for mobile video interpreting will be estimated with hardware depreciation allowances per the US Internal Revenue Code. Costs will be inflation-adjusted to common-year dollars using the Personal Health Care Expenditure Deflator or Personal Consumption Expenditure price index [112].

We will calculate total costs associated with each implementation strategy by summing the above costs. To test *H4a*, we will calculate the ICER for each additional interpreted clinic visit, by calculating the difference in total costs for (i) mobile video interpreting vs education and (ii) mobile video interpreting plus education vs education, and then divide by the difference in number of professionally interpreted visits for providers assigned to (i) mobile video interpreting vs education and (ii) mobile video interpreting plus education vs education. To test *H4b*, we will calculate the ICER for each additional instance of patient comprehension. To do so, we will calculate the difference in total costs for (i) mobile video interpreting vs education and (ii) mobile video interpreting plus education vs education and then divide by the difference in proportion of patients who correctly reported their diagnosis for providers assigned to (i) mobile video interpreting vs education and (ii) mobile video interpreting plus education vs education.

### Regulatory approvals

The mVOCAL Trial was registered on ClinicalTrials.gov on September 22, 2022 (NCT05591586). The Seattle Children’s Hospital institutional review board (IRB) serves as the single IRB (sIRB). The study was initially approved on October 29, 2021 (no. 00003332). All providers and patients will provide informed consent for their participation, with the exception of those participating only through the inclusion of their administrative data, for whom a waiver of informed consent has been obtained.

## Discussion

In this type 3 hybrid implementation-effectiveness study, we will test two discrete implementation strategies for improving professional interpreter use and patient comprehension in primary care. Using a SMART design will allow us to study the effect of the strategies alone and together mirroring the way a practice might implement a staged strategy, with additional intervention for providers with worse performance [64–66]. Given the different barriers targeted by the different strategies, we expect a greater response together, while a single strategy may suffice for many. Our SMART design, mixed methods, and inquiry into mechanisms will illuminate which provider and clinic characteristics would most likely benefit from each strategy, focusing on how, when, where, and why each is effective, rather than simply whether it is effective [115]. As both strategies are inherently scalable but not currently in widespread use, our study will provide actionable data to inform where and how to most effectively implement these strategies to improve safety and equity for patients who use a language other than English for medical care.

We will study these two implementation strategies without additional facilitation, in order to isolate the effect of each, as either could represent the minimum intervention needed to produce change (MINC) [116]. The MINC concept addresses the issue that many effective strategies are not widely adopted due to time and resource limitations in non-research settings. We will therefore test strategies that are relatively simple, with fewer barriers to real-world implementation, as they may lead to greater population impact through wide uptake, even if their individual effect is not as large as might be found for a complex intervention.

With provider-level randomization, contamination between groups is a concern; however, we do not believe it will undermine our ability to test our hypotheses for several reasons. First, we do not expect contamination with the mobile video interpreting strategy, as app access will be controlled by the study team, and we will request that providers not share mobile video interpreting-enabled devices with others. Second, we will measure mobile video interpreting contamination, as every mobile video interpreting use will be linked to a visit via billing invoices, and each mobile video interpreting account will be associated with a specific provider. Mobile video interpreting use at visits with nonmobile video interpreting providers will prompt an inquiry and remediating measures. Third, we will ask providers who are not assigned to the modules not to view them. It is possible that each strategy’s tip sheets may be printed and visible in shared clinic space. However, provider behavior is difficult to change, so we would not expect a minor exposure to meaningfully impact behavior [117]. Finally, we will explore possible contamination in provider qualitative interviews. Evidence of contamination would suggest we should interpret results with caution, but also that the implementation strategy could be widely adopted in practice.

The planned study will generate novel data regarding how effective each strategy is, under what circumstances, through which mechanisms, and at what cost. With these new data, healthcare organizations will be able to make informed decisions to best address the persistent communication-mediated inequities experienced by their patients with language barriers.

## Data Availability

Not applicable.

## Abbreviations

SMART: Sequential multiple assignment randomized trial
MINC: Minimum intervention needed for change
EMR: Electronic medical record
ED: Emergency department
TDF: Theoretical Domains Framework
COM-B: Capability, opportunity, motivation—behavior
PCP: Primary care provider
ICER: Incremental cost-effectiveness ratios
GEE: Generalized estimating equations
HIPAA: Health Insurance Portability and Accountability Act
